# Comparing conventional treatment, single-target rTMS, or dual-target rTMS for the treatment of post-stroke cognitive impairment — clinical effects and neuroscientific insights: study protocol for a randomized controlled trial

**DOI:** 10.1186/s13063-023-07491-x

**Published:** 2023-07-27

**Authors:** Bingshan Xu, Chunrong Lin, Cailian Guo, Hong Wang

**Affiliations:** 1 Present Address: Department of Shanghai Institute of Health Sciences, Shanghai University of Traditional Chinese Medicine, No.1200 Cailun Road, Pudong New Area, Shanghai, 201203 China; 2 School of Exercise and Health, Shanghai University of Sport, Yangpu District, No.399 Changhai Road, Shanghai, 200438 China; 3 College of Rehabilitation Sciences, Shanghai University of Medicine &Health Sciences, No.279 Zhouzhu Road, Pudong New Area, Shanghai, 201318 China

**Keywords:** Double-target, Post-stroke cognitive impairment, Cognitive function, Repetitive transcranial magnetic stimulation

## Abstract

**Background:**

Although increasing evidence suggests that repetitive transcranial magnetic stimulation may help improve cognitive impairment after stroke, its clinical efficacy is still limited. This limitation may be due to the fact that the left dorsolateral prefrontal cortex (DLPFC) is only one of several brain areas involved in post-stroke cognitive impairment (PSCI). The aim of the present study is to reveal whether dual-target stimulation is superior to single-target stimulation and usual care in the treatment of PSCI.

**Methods:**

A single-center, single-blind, randomized controlled trial will be conducted, and fifty-seven PSCI patients will be recruited and randomly assigned to one of three groups based on the stimulating site. The primary outcome is cognitive function, measured using the Montreal Cognitive Assessment Beijing Version (MoCA-BJ) and Mini-Mental Status Examination (MMSE). The secondary outcomes are the modified Barthel Index (MBI), Trail-Making Test (TMT), and digital span test (DST). Furthermore, changes in brain activity are assessed using transcranial Doppler sonography (TCD) examination and serum levels of brain-derived neurotrophic factor (BDNF) and vascular endothelial growth factor (VEGF) closely related to nerve and vascular repair after brain injury. All outcomes will be measured at baseline and 4 weeks after treatment.

**Discussion:**

If dual-target rTMS in significant improvements in cognitive function, this method could be considered as a first-line clinical treatment for PSCI. This proposed study has the potential to identify a new, evidence-based intervention that can enhance cognition and independent living in patients with cognitive impairment after stroke.

**Trial registration:**

Chinese Clinical Trial Registry ChiCTR2200066184. It was registered on 26 November 2022.

## Administrative information

**Table:** 

**Title {1}**	Comparing conventional treatment, single-target rTMS, or dual-target rTMS for the treatment of post-stroke cognitive impairment — clinical effects and neuroscientific insights: study protocol for a randomized controlled trial
**Trial registration {2a and 2b}**	Chinese Clinical Trial Registry ChiCTR2200066184. Registered on 26 November 2022. https://www.chictr.org.cn/ https://www.chictr.org.cn/searchproj.html?title=&officialname=&subjectid=&regstatus=&regno=ChiCTR2200066184&secondaryid=&applier=&studyleader=&createyear=&sponsor=&secsponsor=&sourceofspends=&studyailment=&studyailmentcode=&studytype=&studystage=&studydesign=&recruitmentstatus=&gender=&agreetosign=&measure=&country=&province=&city=&institution=&institutionlevel=&intercode=&ethicalcommitteesanction=&whetherpublic=&minstudyexecutetime=&maxstudyexecutetime=&btngo=btn
**Protocol version {3}**	Version 5 of 2–5-2023
**Funding {4}**	This research is supported by Application of repeated transcranial magnetic stimulation on cognitive function in convalescent patients with stroke(E4-6100–21-201,034)
**Author details {5a}**	Bingshan Xu: ^1^Department of Shanghai Institute of Health Sciences,Shanghai University of Traditional Chinese Medicine, No.1200 Cailun Road, Pudong New Area, Shanghai201203,China Chunrong Lin:^2^School of Exercise and Health, Shanghai University of Sport, No.399 Changhai Road, Yangpu District, Shanghai200438,China Cailian Guo:^2^School of Exercise and Health, Shanghai University of Sport, No.399 Changhai Road, Yangpu District, Shanghai200438,China Hong Wang: ^3^College of Rehabilitation Sciences, Shanghai University of Medicine &Health Sciences, No.279 Zhouzhu Road, Pudong New Area, Shanghai201318,China
**Name and contact information for the trial sponsor {5b}**	Investigator-initiated clinical trial; Hong Wang (Principal Investigator) wanghongplus@163.com *Correspondence: https://www.wanghongplus@163.com
**Role of sponsor {5c}**	This is an investigator-initiated clinical trial. Therefore, the funders plays no role in the design of the study and collection, analysis, and interpretation of data and in writing the manuscript

## Introduction

### Background and rationale {6a}

The Chinese Stroke Report released in 2022 shows that the prevalence rate of stroke in China is 2022.0/100,000, including 1770.7/100,000 for men and 2283.2/100,000 for women [1, 2]. The annual incidence rate is 276.7/100,000, and the mortality is 153.9/100,000. On a global scale, China has become the country with the highest lifelong risk and heaviest disease burden of stroke [3]. Up to one-third of stroke patients will experience post-stroke cognitive impairment [4]. Cognitive impairment is a common and potentially disabling effect of stroke [5]. Post-stroke cognitive impairment (PSCI) is a collective term for differing pathological processes, but regardless of the underlying etiology, stroke survivors and their caregivers consistently rate problems of memory and thinking as their greatest concern [6]. PSCI not only affects the quality of daily life and social adaptability of patients but also has a long-term impact on patients that even exceeds the physical dysfunction itself, seriously affecting the rehabilitation process of patients, and has a high risk of progression to vascular dementia.

Currently, the treatment of PSCI in clinical practice often refers to the treatment of AD, with the principle of improving patient symptoms and delaying disease progression. In addition to medication treatment, rehabilitation training is mainly conducted, but the overall clinical efficacy is unsatisfactory. Rehabilitation training has specific requirements for the patient's limb function. If there is a cognitive impairment, the patient’s ability to understand, execute, and remember will be affected, and they will be unable to manage the training instructions well. Under normal circumstances, the left and right hemispheres of the body are in a balanced state of mutual inhibition. After the stroke, due to the lack of inhibitory effect of the damaged hemisphere on the undamaged hemisphere, it is easy to cause a relative increase in excitability of the intact hemisphere. The inhibitory effect of the damaged hemisphere is exacerbated. Repetitive transcranial magnetic stimulation (rTMS), as a new non-invasive electrophysiological technique, can induce plasticity changes in the central nervous system [7], thereby regulating the excitability of the cerebral hemisphere to change this imbalance after stroke. This study observes the impact of dual-target rTMS on PSCI patients to provide theoretical support for selecting clinical treatment plans.

### Objectives {7}

The objectives of this study are:

1. To examine the efficacy of conventional treatment in improving cognitive function in stroke patients;
2. To evaluate the efficacy of single target high-frequency rTMS stimulation in improving cognitive function in stroke patients;
3. Explore whether the therapeutic effect of dual-target rTMS on cognitive function in PSCI patients is better than that of a single target; and
4. Analyze the mechanism of high-frequency rTMS in improving cognitive impairment after stroke using indicators such as brain Doppler ultrasound and serology.

### Trial design {8}

**Fig. 1:**
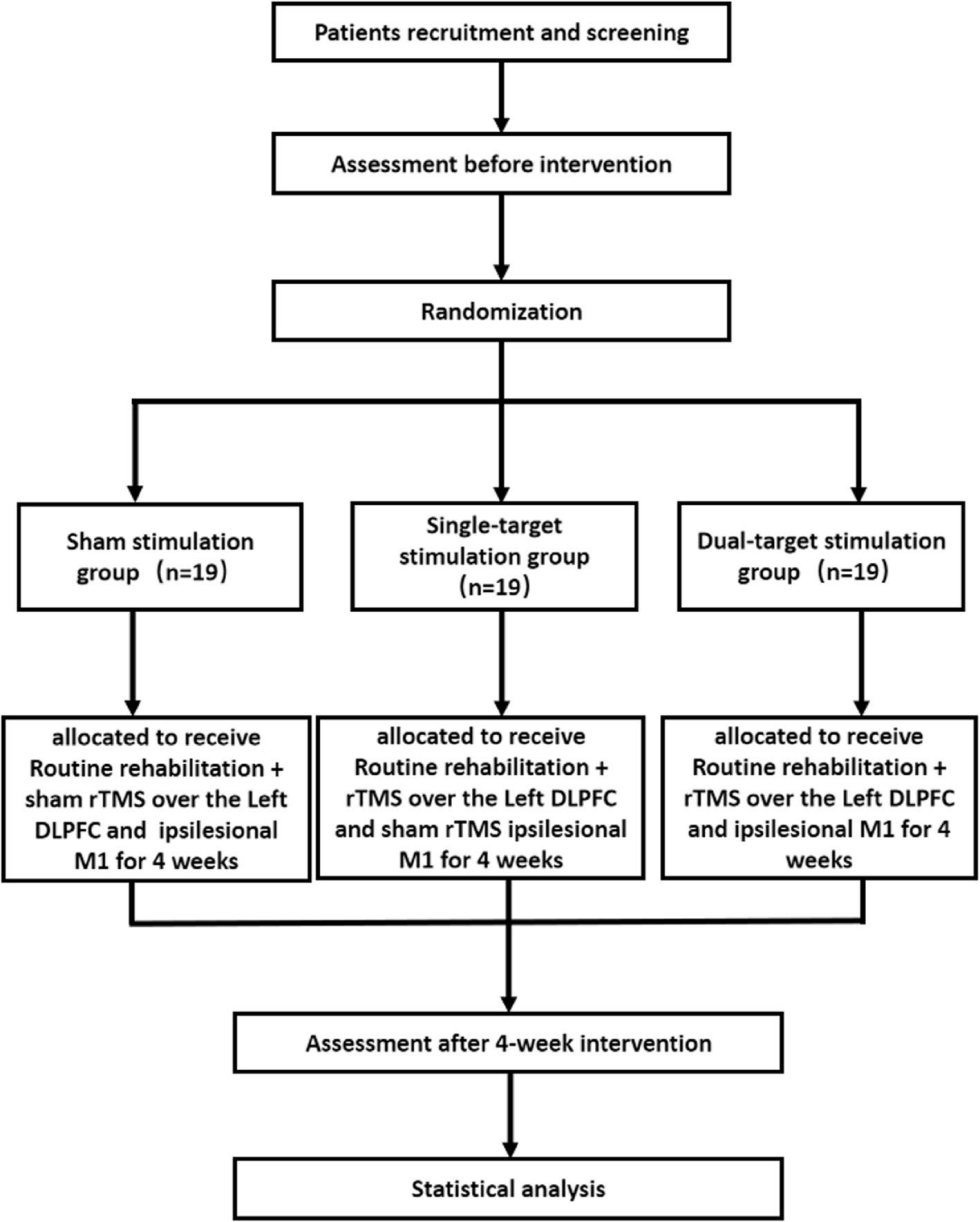
SPIRIT reporting guidelines

This study will include a single-blind, 3-arm, parallel design, randomized superiority controlled trial with a distribution ratio of 1:1:1. Eligible patients will be randomly assigned to the conventional treatment group, single target stimulation group, or dual target stimulation group and will receive intervention treatment for 4 weeks each. This research protocol follows the SPIRIT reporting guidelines [8], as shown in Fig. 1.

## Methods: participants, interventions and outcomes

### Study setting {9}

This study will be conducted at Shanghai Herson Rehabilitation Hospital (Shanghai, China). It is an affiliated hospital of Shanghai University of Medicine &Health Sciences, with a building area of approximately 32,000 m^2^. Approximately 1000 patients with post-stroke cognitive impairment receive treatment annually. Eligibility screening will be shown on patients with post-stroke cognitive impairment in the ward from December 2022 to October 2023; 57 patients will participate in this trial.

### Eligibility criteria {10}

Researchers will conduct eligibility screening based on inclusion and exclusion criteria. All participants should sign an informed consent form after qualification confirmation.

Inclusion criteria:

- Male or female patients between 18 and 75 years of age;
- Right-handed;
- Patients who meet the diagnostic criteria for vascular cognitive impairment reported in the Guidelines from the Vascular Impairment of Cognition Classification Consensus Study [9] and the Chinese vascular cognitive impairment guideline 2019 [10];
- The patient should have a clear consciousness and stable vital signs. They should also be able to express themselves and understand instructions to a certain extent. At least one limb should be movable without any issues, and the patient must be willing to participate in cognitive assessments and related training actively;
- The patient had no cognitive impairment before the onset of the disease but had mild to moderate cognitive impairment after the stroke. They had a primary school education level or above, with a score of 9 < MMSE < 27 and a score of 9 < MoCA < 26;
- No mental illness, no severe aphasia or hearing comprehension disorders; and
- Voluntary participation and signing the informed consent (signed by the patient or the authorized person).

Exclusion criteria:

- Cognitive impairment due to primary or secondary neurological disease, such as normal cranial pressure hydrocephalus, frontotemporal dementia, Parkinson’s disease, multiple sclerosis, encephalitis, and delirium;
- Accompanied with severe mental, intellectual disabilities, or visual and auditory dysfunction, unable to cooperate in completing various functional evaluations;
- Cognitive impairment due to depression, schizophrenia, bipolar disorder, psychotic disorder, vitamin D deficiency, toxicosis, or else systematic diseases;
- Drug/alcohol abuse/dependence within the last 3 months of the first recognition of impairment;
- rTMS treatment contraindications such as epilepsy patients, pregnant or lactating women, or with a metal or electric implanted device (e.g., deep brain stimulator, ventriculoperitoneal shunt, aneurysm clip, pacemaker, cochlear, surgical staples on the scalp);
- MRI contraindications (such as metal implants or claustrophobia); and
- Participating in concurrent pharmacological or nonpharmacologic treatment research.

### Who will take informed consent? {26a}

To determine eligibility for the study, neurologists will evaluate patients who meet the criteria mentioned above. Patients who meet the eligibility criteria will receive initial research details from their doctor and researchers. If they agree to participate, they will be given written information outlining the study's objectives and procedures, and they will sign an informed consent form.

### Additional consent provisions for collection and use of participant data and biological specimens {26b}

The informed consent form indicates that they are asked to cooperate with the investigators for scale assessment, TCD, and blood collection at baseline and after 4 weeks. The patient is informed that all their personal information in the study will be kept strictly confidential. Due to work needs, it shall only be used for the research group members and the research unit's ethics committee. The personal data of these patients will be archived in numbered form, and names and identities will not be exposed or disclosed.

## Interventions

### The explanation for the choice of comparators {6b}

All patients will receive routine rehabilitation treatment (40 min per day, 5 days per week, for 4 weeks), with one-on-one physical and occupational therapy provided by qualified rehabilitation therapists.

### Intervention description {11a}

#### rTMS protocol

The rTMS treatment uses a magnetic stimulator with an 8-shaped coil (MagProX100, Denmark). Participants were divided into three groups: the sham stimulation group, the single-target stimulation group, and the dual-target stimulation group. After recruiting PSCI patients, they were randomly assigned to one of three groups for 20 treatment courses (4 weeks, 5 consecutive days, and 2 days off per week). During the sham stimulation, the “8” shaped coil was placed at a 90° angle to the scalp. The rTMS treatment of the three groups will be completed by a qualified therapist with over 5 years of work experience from the Neurocontrol Room of Shanghai Herson Rehabilitation Hospital.

The detailed information on parameters and grouping were as follows:

- Group 1: sham rTMS over left DLPFC+ ipsilesional M1
- Group 2: left DLPFC+sham rTMS over ipsilesional M1
- Group 3: left DLPFC+ ipsilesional M1
- Left DLPFC: 10 Hz, 80% RMT, 2000 pulses (5s×40 trains,25s interval)
- Ipsilesional M1: 10 Hz, 80% RMT, 1200 pulses, (2s×60 trains, 8s interval)

#### RMT

The resting motor threshold (RMT) is the lowest stimulation intensity to induce at least 50% of the time in a finite number of trials (typically 10).

The RMT was determined as the minimum stimulating power necessary to elicit an overt motor response (motor evoked potential, MEP, more significant than 50 μV) in the contralateral side abductor pollicis brevis (APB) at least 5 of 10 times [11].

#### Localization of stimulation site

The MagPro X100 instrument uses an “8” stimulation coil for transcranial magnetic stimulation in the cortical area, which can collect muscle potential activity. By applying a single pulse stimulation at a higher intensity and moving the coil, the abducts polis MEP > 50 uV can be located and marked as the best stimulation point using a marker. The DLPFC region is targeted for stimulation using the “standard 5 cm” method, which involves translating 5 cm forward from the left M1 region [12].

#### Criteria for discontinuing or modifying allocated interventions {11b}

- Patients who wish to stop participation;
- Patients who could not undergo the baseline assessment;
- Patients who did not complete the rTMS treatment sessions; and
- Patients who suffered from worsening symptoms.

### Strategies to improve adherence to interventions {11c}

To improve the patient's adherence to the intervention protocols, once every rTMS session was completed, a predesigned treatment record card should be filled and signed by the rTMS therapists and the patients or their authorized persons and ultimately returned to the study researchers. The specialized physiotherapists in our center will monitor adherence to the rehabilitation protocol after rTMS. They are in close contact with the treating physiotherapists and monitor progression during study visits.

### Relevant concomitant care permitted or prohibited during the trial {11d}

The route of medical care based on the individualized illness for each patient is permitted, but medications for improving cognition, such as Pfizer’s Aricept, will be prohibited during the trial.

### Provisions for post-trial care {30}

Throughout the entire research process, there is no harm to participants.

### Outcomes {12}

This study measures the effectiveness of rTMS treatment on cognitive ability. Two primary assessments will be conducted, the Montreal Cognitive Assessment Beijing Version (MoCA-BJ) and the Mini-Mental Status Examination (MMSE). In addition, secondary assessments such as the modified Barthel index (MBI), Trail-Making Test (TMT), digital span test (DST), transcranial Doppler (TCD) examination, and serum BDNF and VEGF levels will also be conducted. All assessments will be done twice, pre-treatment (baseline) and post-treatment. A trained neuropsychologist will conduct all cognitive assessments without informing the participants’ cognition status.

1. MoCA-BJ. The MoCA-BJ scale is one of the Chinese versions of the Montreal Cognitive Assessment used to assess general cognitive function [13], which has high sensitivity and specificity for screening cognitive impairment from patients with stroke [14]. The cognitive function was evaluated through alternating connections, drawing cubes, drawing clocks, naming, retelling, subtracting seven from 100, and directional ability. This scale was developed based on MMSE, with a score of 30 points and ≥ 26 points representing normal cognition [15]. A patient’s cognitive function is better if their score is higher.
2. MMSE. The MMSE is a quick and easy cognitive function screening tool commonly used in clinical settings. It takes about 10 min to complete and consists of a single-page, 30-point test that assesses various cognitive domains including memory, attention, and language. A higher score indicates better overall cognitive function, with a score of 27–30 indicating normal function, 21–26 indicating mild impairment, 10–20 indicating moderate impairment, and 0–9 indicating severe impairment [16].
3. TMT(Chinese version). In the TMT-A section, connect the numbers from 1 to 25. TMT-B includes numbers in square and circular shapes, requiring participants to arrange the two forms alternately when connecting numbers in sequence. The scoring criteria are the time taken to complete TMT-A and TMT-B, as well as the interference amount (TMT-B time consumption minus TMT-A time consumption) [17]. This tool is commonly used for diagnosing visual attention, executive function, and processing speed in neuropsychological diseases [18, 19].
4. ADL. The primary activities of daily living are assessed with the modified Barthel Index (MBI) [20], which is used to evaluate individual self-care performance and derived from the Barthel Index. In this study, we used the Chinese version of MBI [21]; it also consists of ten items: personal hygiene, bathing, feeding, toileting, stair climbing, dressing, bowel control, bladder control, ambulation or wheelchair and chair-bed transfer with total 100 scores indicating completed independence. A higher score on the MBI indicates superior independent living ability [22].
5. DST. The scale can be divided into digit span forward (DSF) and digit span backward (DSB), each of which consists of two sets of 2-digit to 10-digit tables. The test begins with item 1 and is stopped after two incorrect attempts. The total score of the DSF and DSB indicates the participant’s attentional functioning, with a higher score indicating better function. A score of less than 5 suggests impaired attention function [23].
6. TCD. According to the latest epidemiological survey, cerebrovascular disease and its risk factors are significant contributors to cognitive decline. By managing these risk factors, it is possible to delay cognitive decline [24]. TCD can assess large vascular lesions and cerebrovascular function, which can provide a basis for early diagnosis and treatment of PSCI [25]. The main indicators for assessment are the average blood flow velocity (Vm), pulsatile index (PI), and resistance index (RI) of the patient’s middle cerebral artery, which measure cerebral hemodynamic changes.
7. Blood sampling. Serum samples will be obtained to analyze BDNF and VEGF levels at baseline and post-treatment. Peripheral venous blood (5 ml) will be collected from 7:00 am to 9:00 am in a climate-controlled room following overnight fasting [26]. The blood was then centrifuged to separate the serum and stored at -80℃ in a refrigerator. Serum levels of BDNF and VEGF will be measured using enzyme-linked immunosorbent assay (ELISA) kits. The steps were followed according to instructions [27]. All samples will be measured by the same technician who is blinded to the patient treatment status. All samples will be evaluated in duplicate and averaged.

### Participant timeline {13}

**Fig. 2:**
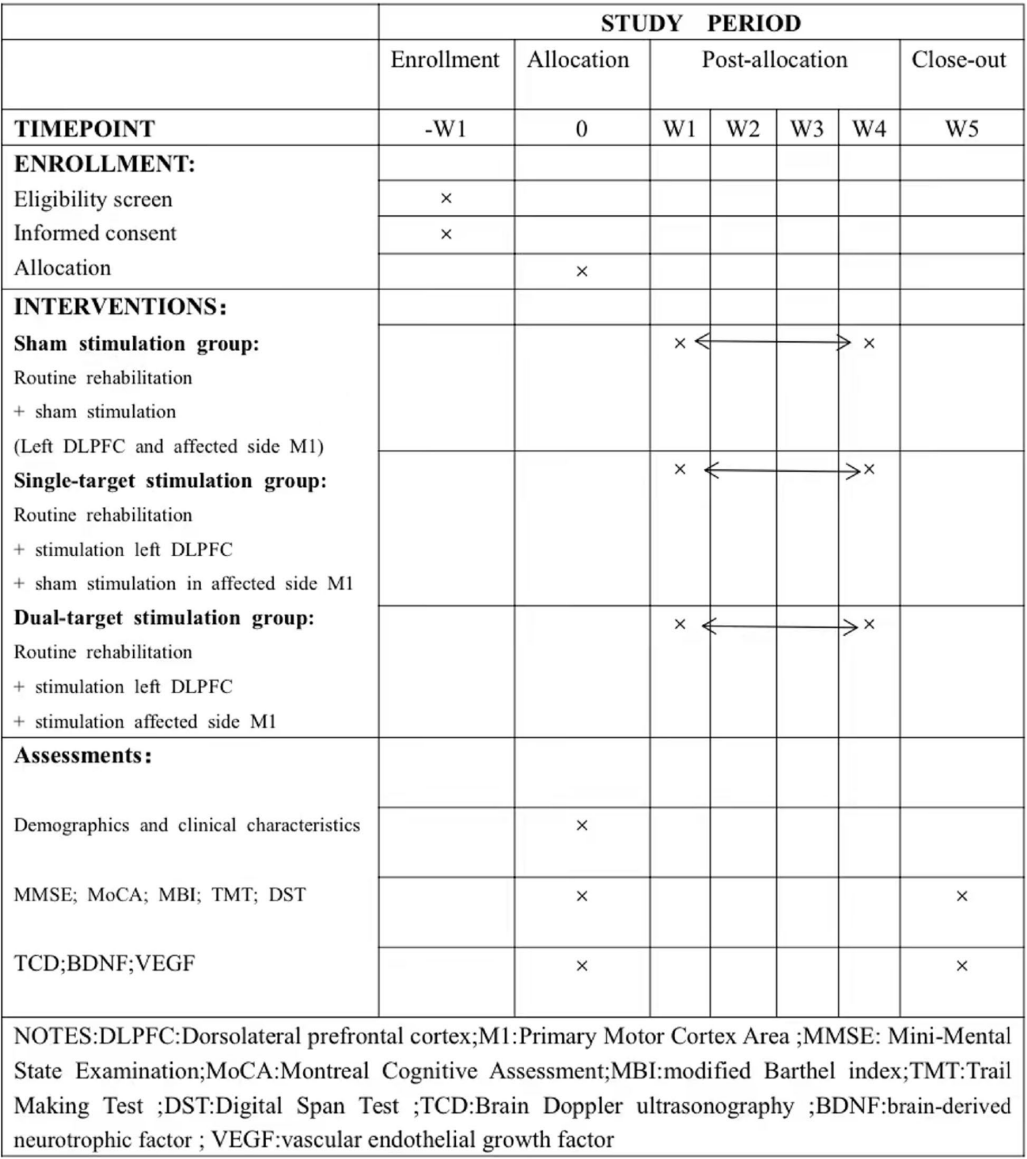
SPIRIT plan

This study will start with the eligibility screening of subjects. Once the eligibility for participation is confirmed, the patient will sign an informed consent form and be randomly assigned to one of the three groups. We will evaluate their clinical scale, perform TCD, and conduct serological tests at the beginning (W0) and after 4 weeks of rTMS treatment (W5). We will keep track of the number of participants who were excluded, refused consent, or withdrew from the study and the reasons why. The SPIRIT plan is shown in Fig. 2.

### Sample size {14}

The study's sample size is based on the expected difference in outcome measure of the MMSE score between the two intervention groups. We determined this effect by reviewing the literature on repetitive transcranial magnetic stimulation's impact on stroke patients [28]. We used a 1:1:1 ratio between groups and calculated the power using G*Power free software (Version 15.0.5). In addition, considering the insufficient sample size caused by patient detachment during the research process, it is necessary to expand the sample size by 20%, resulting in a total sample size of 57 patients.

### Recruitment {15}

This study aims to enroll 57 individuals undergoing rehabilitation at Shanghai Herson Rehabilitation Hospital who have been recommended for rTMS treatment by their doctors or therapists. These individuals must also be willing to participate in the study. Once verbal consent is given to participate in the study, written and oral information regarding the research objectives and procedures will be provided to all participants. All personal information and other data regarding potential or registered patients can be tracked in medical records and obtained from the corresponding authors upon request to protect confidentiality. After receiving written informed consent from qualified screening participants, the study will begin with baseline evaluation and be randomly assigned.

## Assignment of interventions: allocation

### Sequence generation {16a}

To ensure fairness, an independent statistician uses SPSS.25 software to generate randomized allocations for eligible patients. The patients are then evenly distributed into three groups based on their rTMS parameters. The statistician has no involvement in evaluating or executing the experiment.

### Concealment mechanism {16b}

The results of random allocation and detailed rTMS plan are communicated by statisticians by phone to rTMS physical therapists and doctors.

### Implementation {16c}

A statistician uses SPSS.25 software to generate random numbers sorted by size. Numbers 1–19 are for the control group, 20–38 are for the single-target stimulation group, and 39–57 are for the double-target stimulation group. Patients are recommended by rehabilitation doctors, therapists, or any other doctor and voluntarily receive rTMS treatment. The doctor randomly assigns 57 patients to any of the three groups according to the results of the random sequence.

## Assignment of interventions: blinding

### Who will be blinded {17a}

The exact allocation and treatment plan are not disclosed to patients, as well as other researchers such as evaluators and analysts. And rTMS physical therapists are not involved in other research work, such as patient recruitment, randomization, allocation, outcome evaluation, and data analysis.

### Procedure for unblinding if needed {17b}

There will be no need for unblinding throughout the entire research process.

## Data collection and management

### Plans for assessment and collection of outcomes {18a}

This experiment will use a paper case record form (CRF) to record all experimental processes and result in data. To ensure patient privacy, each participant will have a unique identification code. During the registration phase, two data administrators will input the CRF into the computer and check paper and electronic data. The MRIs will be conducted at the Shanghai Herson Rehabilitation Hospital Department of Radiology.

### Plans to promote participant retention and complete follow-up {18b}

During the recruitment process, patients will be provided with comprehensive information about the study, including its setup and requirements. They will also be reminded about the importance of completing the follow-up. Patients can stop at any time during the study and are not obliged to give a reason to discontinue. To assess the patient’s progress, a quality form will be used, and patients will be reminded to cooperate with the researcher’s scale assessment throughout the survey. We will collect any outcome data from participants who have discontinued or deviated from intervention protocols.

### Data management {19}

In this trial, we will use a paper CRF to record all process and outcome data. Each participant will be assigned a unique identification number to protect their privacy. During enrollment, two data managers will input the CRF into the computer and verify both the paper and electronic data. The final trial dataset will be accessible to all study co-applicants and researchers.

### Confidentiality {27}

During the study, research data will be labeled with a unique study identification code for each participant. The key to this identification code list will only be accessible to the research team. After completing the study, the principal investigator will follow research guidelines to document and safeguard the key. No patients identification details will be reported in publications.

### Plans for collection, laboratory evaluation and storage of biological specimens for genetic or molecular analysis in this trial/future use {33}

According to China’s legislation, the leftover material(tested blood samples) will be appropriately treated as medical waste.

## Statistical methods

### Statistical methods for primary and secondary outcomes {20a}

Statistical analysis will be conducted using SPSS version 25.0 and Microsoft Excel 2016 software.

Measurement data (e.g., MoCA BJ, MMSE, TMT, DST, MBI, BDNF, and VEGF levels in serum, TCD) were statistically described by mean ± standard deviation (*x* ± *s*); count data (such as stroke type, hemiplegic side, and stroke risk factors) were described by frequency (*n*) and percentage (%). The measurement data were first tested for normality, and those not conforming to the normal distribution were expressed by M (P25, P75) and analyzed by Kruskal–Wallis H test; the data conforming to the normal distribution were expressed by *x* ± *s*, one-way analysis of variance was used for comparison between multiple groups, LSD-t-test, and paired *t*-test was used for patients before and after treatment. All test levels were significant at *α* = 0.05 and *P* < 0.05.

### Interim analyses {21b}

There are no interim analyses planned.

### Methods for additional analyses (e.g., subgroup analyses) {20b}

There are no subgroup analyses planned.

### Methods in analysis to handle protocol non-adherence and any statistical methods to handle missing data {20c}

We will evaluate the loss of primary outcome data through intention-to-treat analysis. Missing data will be reduced to a minimum by using the multiple imputation.

### Plans to give access to the complete protocol, participant-level data and statistical code {31c}

The corresponding author can make the datasets used and analyzed during the current study available upon reasonable request and in agreement with China’s research collaboration and data transfer guidelines.

## Oversight and monitoring

### Composition of the coordinating center and trial steering committee {5d}

This trial is overseen by a team consisting of a principal investigator who is responsible for both prosecution and medical care of patients, a data manager who ensures data capture and quality, a study coordinator who handles registration and study visits, and a study physician who identifies potential recruits and ensures proper follow-up. The team meets every Thursday, but there is no trial steering committee or stakeholder/public involvement group involved.

### Composition of the data monitoring committee, its role and reporting structure {21a}

To ensure the integrity of the study, a Data Monitoring Committee (DMC) is necessary to maintain the blinding of the researchers and physicians. Additionally, a safety officer with expertise in adverse reactions will be designated to oversee the study’s safety. In the event of any adverse reactions, the safety officer will be notified within 24 h.

### Adverse event reporting and harms {22}

Adverse events (AE) during or ≤ 1 h after the session, including headache, scalp dysesthesia/paresthesia at the stimulation site, muscle pain of temporal or neck muscles, and seizures, will be documented after each treatment session and during the whole treatment period. All adverse events would be reported to the DMC and relevant regulatory bodies. In addition, we will also report specific information on the expectedness, seriousness, severity, and causality of adverse events.

### Frequency and plans for auditing trial conduct {23}

Independent supervisors conduct on-site visits twice a month and check the existence and completeness of investigation documents. In addition, the monitoring personnel conducted the following data checks on 25% of randomly selected patients: informed consent, inclusion and exclusion criteria, source data, missing scales, and reporting.

### Plans for communicating important protocol amendments to relevant parties (e.g., trial participants, ethical committees) {25}

A pre-experiment will be done before the start of the formal experiment, communicating with all relevant parties (e.g., trial participants and ethics committees) about all the problems encountered to ensure no significant or substantial modifications during the trial. If needed, additional consent will be requested and registered. Also, online trial registries will be updated accordingly.

### Dissemination plans {31a}

The results of this research will be entirely disclosed in international peer-reviewed journals. Both positive and negative consequences will be reported.

## Discussion

Currently, the treatment of PSCI in clinical practice often refers to AD, and drug therapy is chosen, but there is no recognized effective treatment drug, and drug therapy mostly has varying degrees of toxic side effects. Research has shown that exercise intervention can delay the decline of cognitive function, and cognitive improvement can also improve memory, understanding, and execution abilities, thereby better conducting motor function training. A multi-component intervention based on exercise combined cognition is more effective than a single intervention and can generate longer-term maintenance benefits [28]. As a new non-invasive green stimulation therapy, repetitive transcranial magnetic stimulation has been clinically proven to improve cognitive impairment after stroke, but it has certain limitations. Traditional rTMS stimulation for cognitive improvement is only applied to a single target brain area, while brain regions are interconnected [29]. In recent years, a study has evaluated the therapeutic effect of dual-target high-frequency rTMS stimulation on the primary motor (M1, bilateral) and left dorsolateral prefrontal cortex (DLPFC) in Parkinson’s disease (PD). The results show that it has a positive therapeutic effect on motor and emotional symptoms of Parkinson’s disease, significantly more robust than the sham stimulation group [30]. ShuqianLi et al. also found that both high-frequency (HF) rTMS and low-frequency (LF) rTMS have therapeutic effects on exercise in PD patients; Stimulation of the primary motor cortex (M1), auxiliary motor area, dorsal prefrontal cortex (DLPFC), and M1 + DLPFC showed therapeutic effects; Stimulating the left DLPFC showed a significant therapeutic impact on PD depression, and HF-rTMS showed a therapeutic effect on depression [31]. PD is a common neurodegenerative disease in clinical practice, with core symptoms such as motor delay, muscle rigidity, static tremor, and a series of non-motor symptoms such as olfactory dysfunction, sleep disorders, cognitive impairment, and autonomic dysfunction. Among them, cognitive impairment is the most common and crucial non-motor symptom. There is a specific correlation with the clinical manifestations of PSCI patients, and PSCI patients also have motor and cognitive impairments. Based on the above research, this study uses dual-target stimulation of brain regions to promote mental function reconstruction, and rTMS sequential stimulation is performed in different brain functional regions.

## Strengths

Based on the bidirectional promotion theory of cognition and movement, that is, cognitive improvement can improve memory, understanding, execution abilities, etc., thereby better conducting motor function training; Improving motor function can also delay the decline of cognitive function. Our study is the first to use left DLPFC combined with M1 as a stimulation target for intervention in PSCI, comparing the efficacy of dual and single targets to improve the effectiveness of PSCI. If the research results are consistent with the expected results, it will provide a new treatment plan for the clinical treatment of post-stroke cognition. Secondly, detecting TCD and serological indicators before and after treatment helps explore the potential mechanisms of rTMS stimulation in improving post-stroke cognition.

## Limitations

Although this study proposes a new rTMS protocol to explore better transcranial magnetic stimulation parameters for the treatment of PSCI, it still has many limitations due to recruitment difficulties in the context of COVID-19 management in 2019, including small sample sizes and single-center studies. In addition, the relatively simple parameter design of PSCI patients and the heterogeneity of oral medication may also be deficiencies in cortical excitability and its response to transcranial magnetic therapy. Multi-center, large sample, and more rigorous design research may be a solution.

## Trial status

This trial was registered on the website of the Chinese Clinical Trial Registry (registration number: ChiCTR2200066184; https://www.chictr.org.cn/). At the writing of this paper, we are recruiting subjects. The study began in December 2022 and is planned to complete in October 2023.

## Data Availability

The datasets used or analyzed during the current study will be available from the corresponding author upon reasonable request. This manuscript does not contain individual personal data from patients.

## Abbreviations

rTMS: Repetitive transcranial magnetic stimulation
PSCI: Post-stroke cognitive stimulation
DLPFC: Dorsolateral prefrontal cortex
RCT: Randomized controlled trial
TCD: Transcranial Doppler sonography
BDNF: Brain-derived neurotrophic factor
VEGF: Vascular endothelial growth factor
ADL: Activities of daily living
MoCA: Montreal Cognitive Assessment Beijing Version
MMSE: Mini-Mental Status Examination
MBI: Modified Barthel Index
TMT: Trail-Making Test
DST: Digital span test
V_m_: Average blood flow velocity
PI: Pulsatile index
RI: Resistance index
ELISA: Enzyme-linked immunosorbent assay
MRI: Magnetic resonance imaging
